# Unlocking the Potential of Poloxamine 908 for Injectable Nanocomposite Hydrogels: Rheological Performance, Dissolution Behavior and Sustained Drug Release

**DOI:** 10.3390/pharmaceutics18101204

**Published:** 2026-09-22

**Authors:** Elena Giuliano, Agnese Gagliardi, Emanuela Longo, Valeria Gaetano, Shabir Ahmad, Donato Cosco

**Affiliations:** 1 Department of Health Sciences, University of Catanzaro “Magna Græcia”, Campus Universitario “S. Venuta”, 88100 Catanzaro, Italy; elena.giuliano@unicz.it (E.G.); gagliardi@unicz.it (A.G.); emanuela.longo@unicz.it (E.L.); valeria.gaetano@unicz.it (V.G.); shabir.ahmad@unina.it (S.A.); 2 “AGreenFood” Research Center, University of Catanzaro “Magna Græcia”, Campus Universitario “S. Venuta”, 88100 Catanzaro, Italy; 3 Department of Chemical, Materials, and Industrial Production Engineering (DICMaPI), University of Naples Federico II, 80125 Naples, Italy

**Keywords:** injectable systems, polymeric nanoparticles, PLGA, zein, rheology, poloxamine 908

## Abstract

**Background/Objectives**: Poloxamine 908 (P908), an amphiphilic block copolymer with thermo- and pH-responsive properties, was demonstrated to be a biomaterial that can be employed for the development of injectable hydrogels. The present study aimed to investigate the influence of nanoparticles with different chemical compositions and surface properties on the properties of P908 hydrogels. **Methods**: Poloxamer 188 (P188)-PLGA, P188-zein and sodium deoxycholate (SD)-zein nanoparticles obtained by nanoprecipitation were incorporated into 30% (*w*/*w*) P908 hydrogels using the cold preparation method. The formulations were characterized by rheological analysis, dissolution studies, FT-IR spectroscopy, in vitro release analysis using rutin as a model compound, and DPPH radical scavenging assay to evaluate the bioactivity of nanoparticles contained within the hydrogel matrix. **Results**: The incorporation of nanosystems preserved the injectability and thermoresponsive behavior of P908 hydrogels and affected their rheological and dissolution properties in different ways. P188-PLGA NPs increased viscosity by approximately 23%, whereas P188-zein NPs caused a slight decrease of approximately 9%, compared with plain P908 at 37 °C and 1 s^−1^. Both formulations showed a moderate increase in T_sol–gel_, reaching 28.5 and 28.8 °C, respectively, compared with 26.4 °C for plain P908 systems. A more pronounced effect was observed with SD-zein NPs, which reduced viscosity by approximately 29% and shifted T_sol–gel_ to 33.9 °C. Moreover, SD-zein NPs enhanced hydrogel dissolution under acidic conditions, with complete dissolution occurring after 6 h at pH 5.0 compared with 8 h at pH 7.0. FT-IR demonstrated the effective inclusion of the colloidal systems within the P908 matrix. Nanocomposite hydrogels provided a more sustained release of rutin with respect to that obtained without the copolymeric matrix, with positively charged P188-zein nanosystems showing an enhanced release at acidic pH, likely due to electrostatic repulsion with the protonated P908 network. DPPH assay confirmed that the inclusion of rutin-loaded nanoparticles into P908 hydrogels preserved the antioxidant activity of the active compound, with a slight increase in radical scavenging capacity, due to the intrinsic mild antioxidant capacity of the copolymeric matrix. **Conclusions**: P908 is a suitable biomaterial to develop nanocomposite hydrogels. The composition and surface properties of nanoparticles allow for the modulation of hydrogel features, enabling the design of therapeutic formulations with tailored rheological properties and characteristics of drug release.

## 1. Introduction

Injectable in situ-forming hydrogels have gained increasing attention as minimally invasive drug delivery systems since they can be administered in liquid form through a conventional needle and subsequently undergo gelation directly at the target site, avoiding the surgical implantation required by preformed matrices [1]. Among the different stimuli that can trigger this sol-to-gel transition, temperature is particularly attractive because it does not require the use of cross-linking agents or external physical treatments that could be harmful to the surrounding tissues [2]. For this reason, thermoresponsive amphiphilic block copolymers based on poly(ethylene oxide) (PEO) and poly(propylene oxide) (PPO) units, such as poloxamers and poloxamines, are among the most widely investigated materials for the development of injectable hydrogels, and their combination with drug-loaded nanocarriers into nanocomposite hydrogels continues to be investigated for a variety of parenteral and topical applications [3,4,5,6,7].

Poloxamine 908 (P908), commercially available as Tetronic^®^ or Synperonic^TM^ 908, is an X-shaped tetrablock copolymer characterized by a high hydrophilic–lipophilic balance (HLB > 24), which self-assembles into micelles and undergoes a reversible sol-to-gel transition at temperatures close to that of the human body [8,9].

Unlike poloxamers, poloxamines contain a central ethylenediamine core with two tertiary amine groups, which confers pH sensitivity in addition to their thermoresponsive behavior. Changes in the protonation state of these groups can affect PEO-PPO hydration and packing, thereby influencing micellization and gelation [5,9].

This dual thermo- and pH-responsive behavior makes poloxamines particularly interesting for the development of drug delivery systems for diseases characterized by dynamic and heterogeneous microenvironments, such as cancer, chronic wounds, inflammatory disorders and tissue degeneration [10].

We have recently demonstrated that P908 aqueous solutions prepared at concentrations ≥ 25% *w*/*w* behave as viscoelastic, shear-thinning systems at 37 °C, with a tunable sol–gel transition temperature depending on the copolymer concentration [8]. Moreover, these hydrogels were able to sustain the release of both a small hydrophilic probe (rhodamine B) and a model protein (ovalbumin) for up to 120 h, while showing negligible cytotoxicity on human keratinocytes [8]. More recently, our research team also provided a first proof of concept of a P908-based multistage system, in which zein nanoparticles (NPs) containing irinotecan and bevacizumab were embedded within the copolymeric matrix for the localized co-delivery of chemotherapeutic and antiangiogenic agents in colorectal cancer: the incorporation of the colloidal systems did not affect the thermogelling behavior of P908 and contributed to modulate drug release and cytotoxicity compared with the free drugs [11].

In addition to P908, other poloxamines have recently been investigated as building blocks for multicomponent systems intended for the delivery of various drugs [6,12,13]. In this context, poloxamines have been combined with cyclodextrins to develop supramolecular systems in which the solubilizing properties of cyclodextrins complement the self-assembling behavior of the copolymer, further increasing the versatility of these platforms for drug delivery [6,12,13]. Poloxamine-based nanocomposite hydrogels embedding Laponite^®^ inorganic NPs have also been described for the sustained release of anticancer compounds, confirming the growing interest in this class of systems [14].

However, formulations specifically combining P908 with biodegradable polymer-based NPs, such as zein- or poly(lactic-co-glycolic acid) (PLGA)-based systems, remain poorly investigated and, to the best of our knowledge, have not yet been systematically characterized.

Zein, a prolamin-rich protein extracted from corn, has emerged as a highly promising raw biomaterial for the development of nanoparticulate carriers, owing to its excellent biocompatibility, biodegradability, amphiphilic structure and inherent mucoadhesive properties, which make it suitable for the encapsulation of both hydrophilic and lipophilic compounds [15,16,17,18]. Alongside natural polymers, synthetic biodegradable materials have also been extensively investigated for the development of polymeric NPs. Among them, poly(lactic-co-glycolic acid) (PLGA) represents one of the most widely used polymers in drug delivery due to its well-established biocompatibility and biodegradability, together with its ability to encapsulate a broad range of active compounds and modulate their release over time [15,19,20]. For these reasons, both zein and PLGA represent attractive biomaterials for the development of nanoparticle-based formulations, offering complementary natural and synthetic approaches for controlled drug delivery.

While our previous investigation demonstrated the potential of P908 for the development of a multistage nanoparticle–hydrogel system for the co-delivery of anticancer agents, the influence of nanoparticle composition and surface properties on the technological behavior of P908-based nanocomposite hydrogels remains largely unexplored. In particular, FT-IR analysis can provide information on the compatibility between the NPs and the copolymeric matrix, while passive microrheology can further clarify the organization and strength of the hydrogel network at rest. Dissolution/erosion studies are also important to understanding the behavior of the formulation over time in biological fluids and its ability to sustain drug release [4,8]. Moreover, considering the intrinsic pH sensitivity of P908, the influence of environmental pH on the nanocomposite matrix and on drug release also requires further investigation. To the best of our knowledge, these aspects have not yet been investigated in P908-based nanocomposite hydrogels containing biodegradable polymeric nanoparticles with different compositions and surface properties.

Based on these considerations, the aim of the present study was to further investigate the suitability of P908 as an injectable dual thermo- and pH-responsive matrix for nanocomposite hydrogels containing biodegradable zein- and PLGA-based nanoparticles. Rutin was selected as a model drug because, in our previous study, its encapsulation and release behavior from the same zein- and PLGA-based nanosystems had already been characterized [15]. The developed formulations were investigated by FT-IR spectroscopy and dynamic and passive rheology, while their dissolution/erosion behavior and rutin-release profiles were evaluated at pH 5 and 7. Finally, the 2,2-diphenyl-1-picrylhydrazyl (DPPH) assay was performed to assess whether the antioxidant activity of rutin was preserved after its encapsulation within nanoparticles and nanocomposites. The overall experimental workflow, from nanoparticle preparation and incorporation into the P908 matrix to the subsequent characterization of the developed formulations, is schematically summarized in Figure S1.

## 2. Materials and Methods

### 2.1. Materials

Poloxamine 908 (trademark Synperonic™ T/908) was a gift from Croda Italiana S.p.A (Pavia, Italy) and used without further purification.

PLGA (75:25, molecular weight 66,000–107,000 Da), zein (CAS number 9010-66-62), sodium deoxycholate monohydrate (SD), rutin hydrate (≥94%), and DPPH were all purchased from Merck (Milan, Italy). Poloxamer 188 (P188) was purchased from BASF (Ludwigshafen, Germany).

Deionized double-distilled water was used throughout the study. All other materials and solvents used, including hydrochloric acid (HCl) used for pH adjustment in the dissolution and release studies, were of analytical grade (Carlo Erba, Milan, Italy).

### 2.2. Development of P908 Nanocomposite Hydrogels

#### 2.2.1. Preparation and Physico-Chemical Characterization of Polymeric Nanoparticles

Three polymeric nanosystems were selected for this study: P188-PLGA nanoparticles, P188-zein nanoparticles and SD-stabilized zein nanoparticles (Table 1).

Zein- and PLGA-based nanoparticles were prepared by nanoprecipitation of preformed polymers in an aqueous medium, according to a procedure previously reported by our group [15,18].

Briefly, zein was dissolved in 3 mL of an ethanol/water mixture (2:1, *v*/*v*), while PLGA was solubilized in 2 mL of acetone at a concentration of 6 mg/mL. Each polymer solution was then added to 5 mL of Milli-Q water containing P188 or SD as stabilizers. P188 was used at concentrations of 2.5% and 2.4% *w*/*v* for zein- and PLGA-based nanoparticles, respectively, whereas SD was employed at 1.25% *w*/*v*. The resulting dispersions were homogenized at 24,000 rpm for 1 min using an Ultra-Turrax T25 homogenizer (IKA^®^ Werke GmbH & Co. KG, Staufen, Germany) and subsequently stirred at 600 rpm for 12 h to promote organic solvent evaporation. The final polymer concentrations were 2 mg/mL for zein nanoparticles and 2.4 mg/mL for PLGA nanoparticles (Table 1).

For the preparation of rutin-loaded formulations, the active compound was dissolved in the organic phase before nanoprecipitation. The rutin concentration employed for each nanosystem was selected according to the optimal physicochemical properties, encapsulation efficiency and stability observed in our previous study [15]. Rutin concentrations of 0.8 and 0.6 mg/mL were used for zein- and PLGA-based nanoparticles, respectively [15].

Before incorporation into the P908 hydrogel, the nanoparticle suspensions were characterized in terms of hydrodynamic diameter, polydispersity index and zeta potential by photon correlation spectroscopy using a Zetasizer Advance Pro instrument (Malvern Panalytical, Malvern, UK) [15]. Measurements were performed on three independently prepared samples, and the results are expressed as the mean ± standard deviation.

The encapsulation efficiency (EE%) of rutin was determined by spectrophotometric analysis according to a previously described procedure and calculated as the percentage ratio between the amount of encapsulated molecule and the amount initially added during nanoparticle preparation [15].

#### 2.2.2. Preparation of Thermosensitive Hydrogels

P908 aqueous solutions were prepared using the cold method [8]. An appropriate amount of copolymer (30% *w*/*w*) was slowly dispersed in distilled water at 4 °C under magnetic stirring to promote the formation of a clear solution.

Similarly, P908-based nanocomposite hydrogels were prepared by adding the copoymer to the NPs.

### 2.3. Fourier Transform Infrared (FT-IR) Spectroscopy

The vibrational spectra of NPs and P908-based formulations, previously lyophilized, were analyzed using a Nicolet™ iS5 spectrometer equipped with an iD7 Attenuated Total Reflectance accessory (Thermo Fisher Scientific Inc., Waltham, MA, USA).

FT-IR spectra were acquired over the wavenumber range of 500–4000 cm^−1^ by recording 64 scans at a resolution of 4 cm^−1^ [4,21].

Spectral data were processed using OMNIC software (version 9.12.1019), and the results were obtained from three independent experiments.

### 2.4. Rheology Studies

The rheological behavior of P908-based hydrogels, either as empty formulations or containing polymeric NPs, was investigated by combining conventional rotational rheology with passive microrheology to assess their macroscopic and microscopic viscoelastic properties.

#### 2.4.1. Oscillatory and Steady-State Measurements

The rheological properties of P908 aqueous solutions were investigated using a Kinexus^®^ Pro rotational rheometer (Malvern Panalytical Ltd., Spectris plc, London, UK) equipped with a cone-plate geometry (40 mm diameter; 2° angle), a temperature-controlling Peltier unit and a solvent trap to prevent water evaporation.

Samples (1 mL) were loaded in the liquid state and allowed to rest for 10 min before each measurement to promote relaxation of any pre-shear induced during loading [22].

Steady-state flow measurements were performed at 4 and 37 °C over a shear-rate range of 0.1–100 s^−1^ to evaluate the viscosity and shear-thinning behavior of the systems [23].

Oscillatory measurements were carried out at 37 °C on hydrogels formed directly on the plate of the rheometer. The storage modulus (G′), loss modulus (G″) and phase angle (δ) were evaluated. Frequency sweep measurements were then performed from 0.1 to 10 Hz at a constant stress of 1 Pa, selected within the linear viscoelastic region (LVR) on the basis of our previous rheological characterization of P908-based hydrogels [8].

The solution-to-gel transition temperature (T_sol–gel_) was determined by monitoring G′, G″, and δ from 10 to 50 °C at a heating rate of 1 °C/min, a frequency of 1 Hz and a stress of 1 Pa. T_sol–gel_ was identified as the temperature at which G′ became higher than G″, indicating the transition from predominantly viscous to predominantly elastic behavior [24].

The obtained data were analyzed using rSpace software (Version 1.60).

#### 2.4.2. Diffusing Wave Spectroscopy

The microrheological properties of P908 aqueous solutions were investigated by multi-speckle diffusing wave spectroscopy (MS-DWS) using a Rheolaser^®^ Master instrument (Formulaction, Toulouse, France), as previously reported [8].

Samples, maintained in the liquid state at the storage temperature, were transferred into 20 mL vials and immediately placed in the instrument chamber pre-equilibrated at 37 °C.

Particle dynamics were monitored through changes in the backscattered speckle pattern, and the mean square displacement (MSD) was calculated according to the Weitz and Pine theory to describe the viscoelastic behavior of the systems [25,26]. High particle mobility is characteristic of predominantly liquid-like formulations, whereas restricted motion and the occurrence of an MSD plateau indicate the formation of a viscoelastic network [25,26].

The Solid–Liquid Balance (SLB) and Elasticity Index (EI) were derived from the MSD curves using RheoSoft Master™ software, version 1.3.2.0 (Formulaction, Toulouse, France). SLB values below 0.5 indicate predominantly solid-like behavior, whereas values above 0.5 are associated with liquid-like systems [8]. The EI provides an indication of sample elasticity, with lower MSD plateau values corresponding to a stronger and more elastic polymeric network [25].

### 2.5. In Vitro Hydrogel Dissolution and Rutin-Release Studies

The dissolution/erosion rate of P908-based hydrogels was evaluated in vitro according to the gravimetric method, as previously described [8,27].

A total of 1 mL of each formulation was placed into a pre-weighed vial and equilibrated at 37 °C until a clear gel was formed. Subsequently, 2 mL of dissolution medium, previously equilibrated at 37 °C, was carefully added onto the hydrogel surface. At specific incubation times, the medium was withdrawn, the vials containing the residual hydrogel were weighed, and fresh medium was added to the systems. The amount of dissolved/eroded gel was calculated from the differences in vial weight and expressed as a percentage. The whole experiment was carried out at 37 °C.

The release profile of rutin from nanocomposite hydrogels was assessed by means of the dialysis method [15,21]. The formulations were placed into cellulose acetate dialysis tubing with a molecular-weight cut-off of 12–14 kDa (Spectra/Por^®^, Spectrum Laboratories Inc., Rancho Dominguez, CA, USA) and immersed in the release medium (pH 5 or pH 7), constantly stirred and maintained at 37 ± 0.1 °C. At predetermined incubation times, aliquots of the medium were withdrawn and replaced with an equal volume of fresh medium. The amount of released rutin was determined spectrophotometrically and expressed as a percentage of the drug initially encapsulated within the nanoparticles [15]. For each formulation and sampling time, the corresponding drug-free system subjected to the same experimental conditions was used as a blank to account for any possible absorbance contribution from the formulation components.

Both dissolution/erosion and release experiments were performed at pH 7.0 and 5.0 (adjusted by addition of HCl) in order to investigate the influence of pH on the behavior of the P908-based systems, considering the pH-sensitive properties of the poloxamine [9]. These values were selected to compare a physiological condition with a more acidic environment, representative of the pH variations often considered in smart and bioresponsive drug delivery systems designed for pathological conditions characterized by local acidification, such as tumors, inflammatory disorders, and some diabetes-related tissue alterations [10].

The cumulative rutin-release profiles obtained from the P908-based nanocomposite hydrogels were analyzed according to zero-order, first-order, Higuchi, Korsmeyer–Peppas and Hixson–Crowell mathematical models. The best-fitting model was identified on the basis of the highest correlation coefficient (R^2^) [28].

### 2.6. DPPH Radical Scavenging Activity

Considering the well-known antioxidant properties of rutin, its radical scavenging activity was evaluated using the DPPH assay [29,30].

Free rutin, rutin-loaded zein- and PLGA-based nanoparticles, and the respective nanocomposite hydrogels were evaluated under the same experimental conditions.

Briefly, a 0.1 mM DPPH solution was prepared in ethanol and 100 μL of each sample was mixed with an equal volume of the DPPH solution in a 96-well microplate. After incubation for 30 min at room temperature in the dark, absorbance was measured at 517 nm using a Varioskan LUX plate reader (Thermo Fisher Scientific, Milan, Italy). Radical scavenging activity was expressed as the percentage decrease in absorbance relative to the untreated DPPH control. An appropriate solvent blank was used for each sample [29,30].

### 2.7. Statistical Analysis

Data were expressed as the mean ± standard deviation (SD) of independent experiments. Unless otherwise specified, statistical differences among groups were evaluated by one-way analysis of variance (ANOVA), and *p*-values < 0.05 were considered statistically significant. Normality was assessed by Shapiro–Wilk test.

## 3. Results and Discussion

Smart drug delivery platforms able to respond to different physiological conditions are increasingly investigated for the development of tailored therapeutic formulations [10]. In this context, nanocomposite hydrogels offer the possibility of modulating formulation performance through both the properties of the hydrogel matrix and those of the incorporated nanocarriers [1,10,31]. Accordingly, three nanoparticle formulations previously developed and characterized by our team (P188-PLGA, P188-zein and SD-zein NPs) were included in P908 hydrogels. The selected nanosystems allowed for the comparison of synthetic and natural polymeric matrices, as well as the evaluation of the influence of different stabilizers and surface properties on the technological behavior of the resulting formulations.

The physicochemical characteristics of the drug-free nanoparticles are summarized in Table S1. P188-PLGA NPs showed a mean diameter of ~120, a narrow size distribution and a zeta potential of ~−22 mV. P188-zein NPs were characterized by similar properties, but a positive surface charge was obtained. Conversely, SD-zein NPs displayed a mean diameter of 110 ± 1 nm, a PDI of 0.11 ± 0.01 and a zeta potential of −33 ± 1 mV.

P908 was directly dispersed in the aqueous suspensions of the polymeric NPs at a final copolymer concentration of 30% *w*/*w*. This concentration was selected on the basis of our previous findings demonstrating suitable rheological, thermogelling and dissolution properties for the development of injectable P908-based hydrogels [8]. The plain P908 formulation was prepared under the same conditions, using water as the dispersion medium. As shown in Figure 1A, both plain P908 and the nanocomposite systems maintained a uniform appearance in the liquid state at the storage temperature and after gel formation at 37 °C, suggesting that nanoparticle incorporation did not visibly compromise the thermoresponsive behavior of the polymeric matrix.

### 3.1. FT-IR Spectroscopy

Following the macroscopic evaluation, FT-IR spectroscopy was performed to investigate the inclusion of the nanoparticles within the P908 matrix and possible interactions among the formulation components (Figure 1B). The spectrum of the plain P908 hydrogel showed the characteristic bands of this type of copolymer at approximately 2880 cm^−1^, associated with C–H stretching, and at 1460, 1340 and 1100 cm^−1^, with the latter mainly related to the C–O–C stretching of the PEO/PPO chains [29,32]. The spectra of the nanoparticles showed additional characteristic signals related to their composition. In particular, P188-PLGA nanoparticles demonstrated the typical ester carbonyl band of PLGA at approximately 1760 cm^−1^, whereas P188-zein nanoparticles showed the characteristic amide I band of the protein at around 1640 cm^−1^ [33,34,35,36]. The SD-zein formulation displayed a broader spectral profile, with bands at approximately 3330 cm^−1^, 2930 and 2860 cm^−1^, together with the characteristic amide I and amide II signals at around 1640 and 1550 cm^−1^, respectively [18,37,38].

After incorporation of the NPs into the P908 hydrogel, the spectra of all nanocomposite formulations were mainly dominated by the characteristic bands of the copolymer. The specific signals of PLGA and zein became less evident or were overlapped by the P908 fingerprint, while no new bands or relevant shifts were observed. These findings are consistent with the effective inclusion of the nanoparticles within the P908 network and suggest that their incorporation did not induce detectable chemical modifications of the copolymeric matrix [4].

Comparable spectral behavior has previously been reported for the structurally related thermosensitive copolymer Poloxamer 407 (P407) [4]. Unlike P908, which contains a central ethylenediamine core connected to PEO and PPO blocks, P407 is a linear PEO–PPO–PEO triblock copolymer [39,40,41,42]. Nevertheless, both copolymers undergo temperature-dependent micellization and gelation and can be used to develop injectable thermosensitive hydrogels [7,40]. In P407-based nanocomposite hydrogels, the FT-IR spectra were mainly dominated by the characteristic bands of the copolymer, with only minor variations observed after nanocarrier incorporation [4].

### 3.2. Rheological Characterization

The rheological properties of the P908-based formulations were investigated to establish whether the incorporation of the different nanoparticle formulations affected the flow behavior, mechanical properties and thermoresponsive behavior of the copolymeric matrix. This aspect is particularly relevant for injectable in situ-forming hydrogels, which should combine low viscosity under storage and administration conditions with the ability to rapidly gel at physiological temperature [43,44].

#### 3.2.1. Dynamic Rheological

The presence of nanoparticles induced slight variations in the viscosity of the P908 matrix, which were more evident at 37 °C and depended on the composition of the incorporated nanosystems (Figure 2). P188-PLGA NPs slightly increased the viscosity of the hydrogel, whereas a moderate reduction was observed following the incorporation of P188-zein NPs, with a more pronounced decrease in the presence of SD-zein systems.

Comparable effects of nanocarrier incorporation on the rheological behavior of thermoresponsive poloxamer-based hydrogels have been previously reported. Marques et al. observed an increase in viscosity at room temperature but a reduction at 37 °C, which was attributed to interactions between the nanostructured lipid carriers and P407 micelles during gel formation [3]. Similarly, Pradines et al. reported a reduction in the rheological properties after incorporation of poly(isobutylcyanoacrylate) nanoparticles into P407 hydrogels; microcalorimetric analysis also revealed a decrease in the enthalpy of P407 micellization, suggesting that part of the copolymer interacted with the nanoparticle components rather than participating in conventional micelle formation [45]. These findings demonstrate that nanocarrier composition and the resulting interactions with the copolymeric matrix can differently affect micellar organization and, consequently, the rheological properties of thermoresponsive hydrogels.

Despite these differences, all formulations maintained the characteristic temperature-dependent rheological behavior of P908 (Figure 3) [8,9]. At 4 °C, viscosity remained almost constant over the investigated shear-rate range, confirming that both the plain hydrogel and the nanocomposite formulations behaved as low-viscosity fluids under storage conditions. Conversely, at 37 °C, all systems showed a marked increase in viscosity together with a progressive decrease in viscosity as the shear rate increased, indicating a useful non-Newtonian shear-thinning behavior [46,47,48]. This transition is consistent with the thermoresponsive behavior previously described for P908 aqueous solutions at concentrations ≥ 25% *w*/*w*, in which heating promotes micellization and subsequent intermicellar packing, leading to the formation of a structured gel network [8,9].

The reduction in viscosity under increasing shear is particularly advantageous for injectable formulations, since it facilitates flow through the needle while allowing the system to recover a highly viscous state after administration [49,50].

The oscillatory measurements further highlighted differences in the organization of the hydrogel network (Figure 4). At 37 °C, the storage modulus (G′) remained higher than the loss modulus (G″) throughout the investigated frequency range for all formulations, while the phase angle remained well below 45°. These features indicate a predominantly elastic, solid-like behavior and confirm the formation of a stable viscoelastic network [51,52]. A comparable mechanical spectrum has previously been reported for P908 hydrogels containing at least 25% *w*/*w* copolymer, for which G′ was only weakly dependent on frequency and remained higher than G″ over the entire frequency range [24].

The incorporation of P188-PLGA and P188-zein NPs did not compromise this characteristic mechanical response. Indeed, their G′ values were comparable to those of the plain P908 matrix, indicating preservation of the elastic network. On the contrary, the SD-zein formulation showed lower G′ and G″ values, although G′ remained predominant over G″. This result indicates the formation of a less rigid network rather than the loss of gel-like behavior and is consistent with the lower viscosity observed for the same formulation in the steady-state measurements (Figure 4).

The different response of the three nanocomposite systems suggests that the effect of nanoparticle incorporation cannot be attributed exclusively to the presence of a dispersed nanoparticulate phase but depends on the composition and physicochemical characteristics of the incorporated components. This interpretation is supported by a recent study from our group in which zein nanoparticles stabilized with DSPE-PEG2000 were incorporated into a 25% *w*/*w* P908-based hydrogel [11]. In that case, empty nanoparticles did not substantially modify the viscosity profile of P908, whereas rheological changes became evident when irinotecan hydrochloride, a chemotherapeutic agent, or bevacizumab, an antiangiogenic monoclonal antibody, were entrapped within the nanocarriers. Notably, although the zein matrix and PEG-based stabilizer remained unchanged, the nature of the incorporated molecule was sufficient to differently affect the viscosity and the mechanical properties of the hydrogel, indicating that even slight changes in the composition of the incorporated formulation can modify its interactions with the P908 chains and, consequently, the rheological response of the matrix [11].

A similar dependence on nanocarrier composition was observed in another recent study, where P407 hydrogels were combined with liposomes differing in lipid composition, surface charge and PEGylation [4]. Liposomes based on Phospholipon 90G (PL90G) or 1,2-dipalmitoyl-sn-glycero-3-phosphocholine showed lower physical stability after incorporation into the hydrogel, whereas vesicles containing the cationic lipid 1,2-dioleoyl-3-trimethylammonium-propane, the anionic lipid 1,2-dipalmitoyl-sn-glycero-3-phosphatidylserine, or the PEGylated phospholipid DSPE-mPEG2000 resulted in more stable nanocomposite formulations [4].

Temperature sweep experiments were performed to identify possible variations in the sol–gel transition temperature following nanoparticle incorporation (Figure S2). As expected, the plain 30% *w*/*w* P908 formulation underwent the transition from G″ > G′ to G′ > G″ at approximately 26.5 °C, in agreement with previous findings for the same copolymer concentration [8]. The incorporation of P188-PLGA and P188-zein NPs produced only a moderate shift in the transition toward higher temperatures, whereas a more pronounced increase was observed for the SD-zein formulation (Table 2). Nevertheless, gelation remained below 37 °C in all cases, confirming that nanoparticle incorporation did not compromise the ability of P908 to form a gel under physiological conditions. The higher value observed for the SD-zein system is also consistent with its lower viscosity and reduced elastic modulus, confirming that this formulation interfered more markedly with the temperature-induced organization of the P908 micelles.

A similar influence of nanocarrier incorporation on the gelation temperature has been reported for other nanocomposite-based poloxamer hydrogels. Marques et al. observed an increase in the sol–gel transition temperature of P407 hydrogels following the incorporation of nanostructured lipid carriers, suggesting that the nanocarriers affected the organization and packing of the poloxamer micelles [3]. Conversely, Nie et al. described a progressive decrease in the gelation temperature of F127 hydrogels with increasing liposome concentration [53]. These findings indicate that the effect of nanocarriers on thermoresponsive gelation may vary depending on their composition, concentration and interactions with the copolymeric matrix, consistently with the different shifts in T_sol-gel_ observed for the P908-based formulations investigated in the present study.

#### 3.2.2. Passive Microrheology

Passive microrheology was successively employed to obtain complementary information on the development of the P908 network at rest, without applying an external mechanical perturbation [25,54]. The MSD profiles clearly reflected the temperature-induced evolution of the formulations from the liquid to the gel state (Figure 5).

At the beginning of the analysis, when the samples were transferred directly from the storage temperature to 37 °C, all formulations showed higher MSD profiles, due to a relatively high mobility of the scattering particles. With increasing incubation time at 37 °C, particle motion became progressively restricted and the MSD curves developed a plateau, reflecting confinement of the particles within the increasingly structured polymeric network [55]. This behavior has previously been described for P908 hydrogels and is associated with progressive micellar organization and formation of the three-dimensional gel structure [8,9].

The results obtained by dynamic rheology were further confirmed by the Elasticity Index (EI) and Solid–Liquid Balance (SLB) values (Figure 6). In all formulations, EI progressively increased during incubation at 37 °C and reached a plateau, confirming the formation of an elastic network. Higher EI values were observed for the P188-PLGA and P188-zein systems, whereas the SD-zein formulation showed the lowest values, indicating a weaker network organization (Figure 6). Consistently, SLB progressively decreased below 0.5 in all formulations, confirming the transition toward a predominantly solid-like state during gel formation (Figure 6) [56,57].

Taken together, passive and dynamic rheological analyses showed that NPs modulated the strength and organization of the P908 hydrogel network in a composition-dependent manner, without compromising thermoresponsive gelation at 37 °C.

### 3.3. In Vitro Dissolution and Drug Release Profiles

The gravimetric method was used to evaluate whether the nanosystems influenced the dissolution behavior of the copolymeric matrix in an aqueous environment [58]. This parameter is particularly relevant for in situ-forming systems, since hydrogel dissolution contributes to determining their residence time at the administration site and, consequently, may influence the release kinetics of the encapsulated drug [58].

At pH 7.0, the plain P908 hydrogel showed a progressive dissolution profile, reaching approximately 50% dissolution after 6 h and complete dissolution within 12 h (Figure 7). This behavior is consistent with that previously reported for 30% *w*/*w* P908 hydrogels, which undergo relatively rapid dissolution in aqueous media, related to the highly hydrophilic nature of P908 (HLB > 24) [8,9].

The incorporation of P188-PLGA NPs did not substantially modify the dissolution profile of the P908 matrix, as shown by the close correspondence between the two curves throughout the experiment. Relatively similar behavior was also observed for the P188-zein nanocomposite, although a moderate increase in dissolution became evident at the later time points, with approximately 58% and 81% of the formulation dissolved after 6 and 8 h, respectively (Figure 7).

A markedly different behavior was observed for the formulation containing SD-zein NPs. In this case, dissolution was faster from the early stages, reaching complete dissolution after 8 h (Figure 7). This trend was consistent with the rheological results, since the SD-zein formulation showed the lowest viscosity and viscoelastic moduli, together with a weaker elastic network.

Considering the pH-sensitive nature of poloxamines, the dissolution behavior of the formulations was also evaluated at pH 5.0 (Figure 8).

P908 is characterized by a central ethylenediamine moiety containing two tertiary amine groups, with reported pKa values of approximately 5.2 and 7.9. Consequently, changes in environmental pH can modify the protonation state of the copolymer and, in turn, its self-assembly behavior [9]. At pH 7.0, the central diamine is predominantly monoprotonated, whereas lowering the pH to 5.0 favors a higher degree of protonation. The resulting increase in positive charge enhances copolymer hydration and electrostatic repulsion, making micellar association and subsequent gel organization less favorable. Previous studies on P908 showed that acidic conditions shift micellization and gelation toward higher temperatures and, under strongly acidic conditions, may even suppress gel formation [9].

Consistent with this pH-dependent behavior, dissolution was faster at pH 5.0 than at pH 7.0. For plain P908, as well as for the P188-PLGA and P188-zein nanocomposite hydrogels, dissolution was only slightly faster at pH 5.0, whereas a more pronounced pH effect was observed for the SD-zein formulation, which completely dissolved after 6 h at pH 5.0 (Figure 8).

Overall, the dissolution experiments demonstrate that the P908 matrix preserved its characteristic aqueous solubility after NP incorporation, while the degree of hydrogel erosion could be modulated by both nanocarrier composition and environmental pH.

Rutin was selected as a model compound to evaluate the influence of NP incorporation into the P908 matrix on drug release. As previously reported, this choice was based on our previous study, in which rutin was successfully encapsulated in the same P188-PLGA, P188-zein and SD-zein nanoparticles and its release behavior from the colloidal systems was extensively characterized [15].

The results obtained are consistent with our previous findings, in which rutin-loaded PLGA- and zein-based nanoparticles showed a sustained and prolonged release of the active compound as a consequence of its retention within the polymeric matrix (Figure 9). The incorporation of these nanosystems within the P908 matrix decreased the leakage of rutin compared with the nanoparticle suspensions, demonstrating the additional contribution of the thermosensitive hydrogel to the control of drug diffusion. A similar effect was recently observed for other nanocomposite thermosensitive hydrogels, where the hybrid formulations showed a slower release than the nanocarriers alone [4,53,59].

Moreover, a slightly higher release was generally observed from the nanocomposite hydrogels at pH 5.0 than at pH 7.0, consistently with the faster dissolution of the P908 matrix under acidic conditions (Figure 9). This pH-dependent behavior may be particularly relevant for personalized medicine, since acidic microenvironments are characteristic of several pathological conditions and could therefore favor a more responsive drug release according to the local environment [60,61,62].

To further characterize the release behavior of the nanocomposite hydrogels, the experimental profiles were fitted to different kinetic models. As reported in Tables S2 and S3, the Higuchi model provided the highest R^2^ values for all the investigated formulations at both pH values, suggesting that diffusion played an important role in rutin release under the investigated experimental conditions. This behavior is consistent with previous studies on poloxamer-based hydrogels evaluated using dialysis methods, in which the membrane limits hydrogel dissolution and consequently emphasizes drug diffusion through the polymeric matrix [28,63].

Overall, the present results confirm that the combination of polymeric NPs with the P908 hydrogel provides an additional level of control over the release of encapsulated compounds compared with the nanosystems alone.

### 3.4. Antioxidant Activity Evaluation

Another crucial aspect to be investigated is the influence of the incorporation of the polymeric NPs into the copolymeric matrix on the biological activity of the entrapped drugs. For this reason, considering the well-known antioxidant properties of rutin, the radical scavenging activity of the different formulations was evaluated by means of the DPPH assay [28,30].

As expected, free rutin showed concentration-dependent radical scavenging activity, and a similar trend was observed after its encapsulation within the different nanoparticle formulations, indicating that the preparation process did not compromise the antioxidant properties of the flavonoid (Figure 10). Interestingly, the incorporation of the nanoparticles into the P908-based hydrogel generally resulted in further enhancement of the DPPH scavenging activity, which was also observed for the corresponding empty systems (Figure S2). This effect is probably attributable, at least in part, to the intrinsic radical scavenging activity of the copolymeric matrix (Figure S3), as previously observed for P407-based hydrogels [27,28]. Although the exact mechanism was not fully elucidated, this behavior may be related to the amphiphilic PEO–PPO–PEO structure of the copolymers and to the large number of oxyethylene groups, which are able to establish intermolecular interactions and influence hydrogen transfer processes involved in the DPPH reaction [28].

## 4. Conclusions

The obtained results support the feasibility of combining different polymeric nanoparticles with a P908-based hydrogel to obtain in situ-forming nanocomposite formulations able to provide a prolonged and sustained release of active compounds, preserving their bioactivity.

Rheological characterization showed that nanoparticles did not compromise the thermosensitive and viscoelastic behavior of the P908 matrix; however, the presence and composition of the nanocarriers promoted changes in viscosity. These features are particularly relevant because they modulated the dissolution and drug release profiles of the formulations. Moreover, these parameters were affected by pH, showing that the systems may respond differently depending on the surrounding microenvironment. Such pH-dependent modulation could represent an additional advantage for the development of more adaptable and potentially personalized therapeutic strategies. In particular, their dual-responsive behavior may favor their application in pathological conditions characterized by dynamic and heterogeneous pathophysiological environments, including variations in temperature and pH. These features may be particularly relevant for solid tumors, chronic wounds, inflammatory diseases and tissue regeneration, where local administration and environment-responsive drug release could improve the therapeutic response [60,61,62].

Overall, the results demonstrate the versatility of P908 hydrogels as systems capable of incorporating various polymeric nanoparticles, maintaining suitable physicochemical and functional properties. Nevertheless, the present work should be considered a preliminary proof-of-concept study. In particular, it was mainly focused on an initial comparison of P908-based nanocomposite hydrogels containing polymeric nanoparticles made up of different biomaterials and stabilizers. Future studies should therefore evaluate the impact of various colloidal concentrations in order to better understand their influence on the properties of P908-based nanocomposite systems. Long-term stability studies should also be performed to assess whether the physicochemical characteristics of the embedded nanocarriers and the rheological properties of the injectable formulations are maintained over time. Moreover, additional in vitro studies addressing cytocompatibility and biological performance, together with in vivo investigations, will be necessary to evaluate whether the sustained release behavior is preserved after administration, as well as local tolerability, safety, biodegradation, residence time and therapeutic efficacy. Finally, the use of therapeutically relevant active compounds will be important to confirm the applicability of these nanocomposite systems for specific pathological conditions.

## Figures and Tables

**Figure 1:**
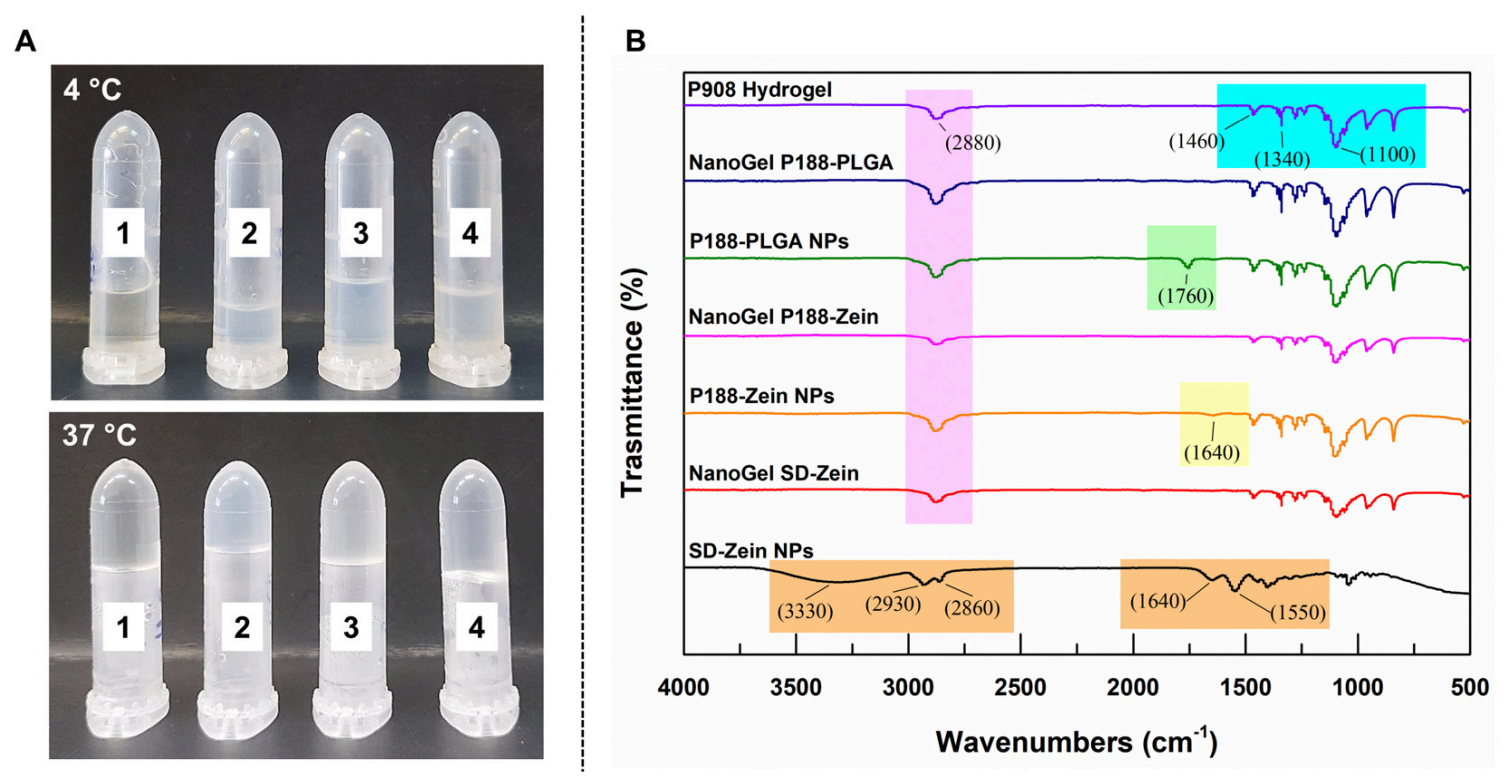
(**A**) Representative photographs of plain poloxamine 908 P908 (1) and P908 formulations containing P188-PLGA (2), P188-zein (3) or SD-zein (4) nanoparticles at 4 °C and after gel formation at 37 °C. (**B**) FT-IR spectra of the plain P908 hydrogel, the nanoparticle formulations (NPs) and the corresponding nanocomposite hydrogels (NanoGels). P188, Poloxamer 188; PLGA, poly(lactic-co-glycolic acid); SD, sodium deoxycholate. The colored shaded areas indicate the spectral regions corresponding to the main characteristic absorption bands.

**Figure 2:**
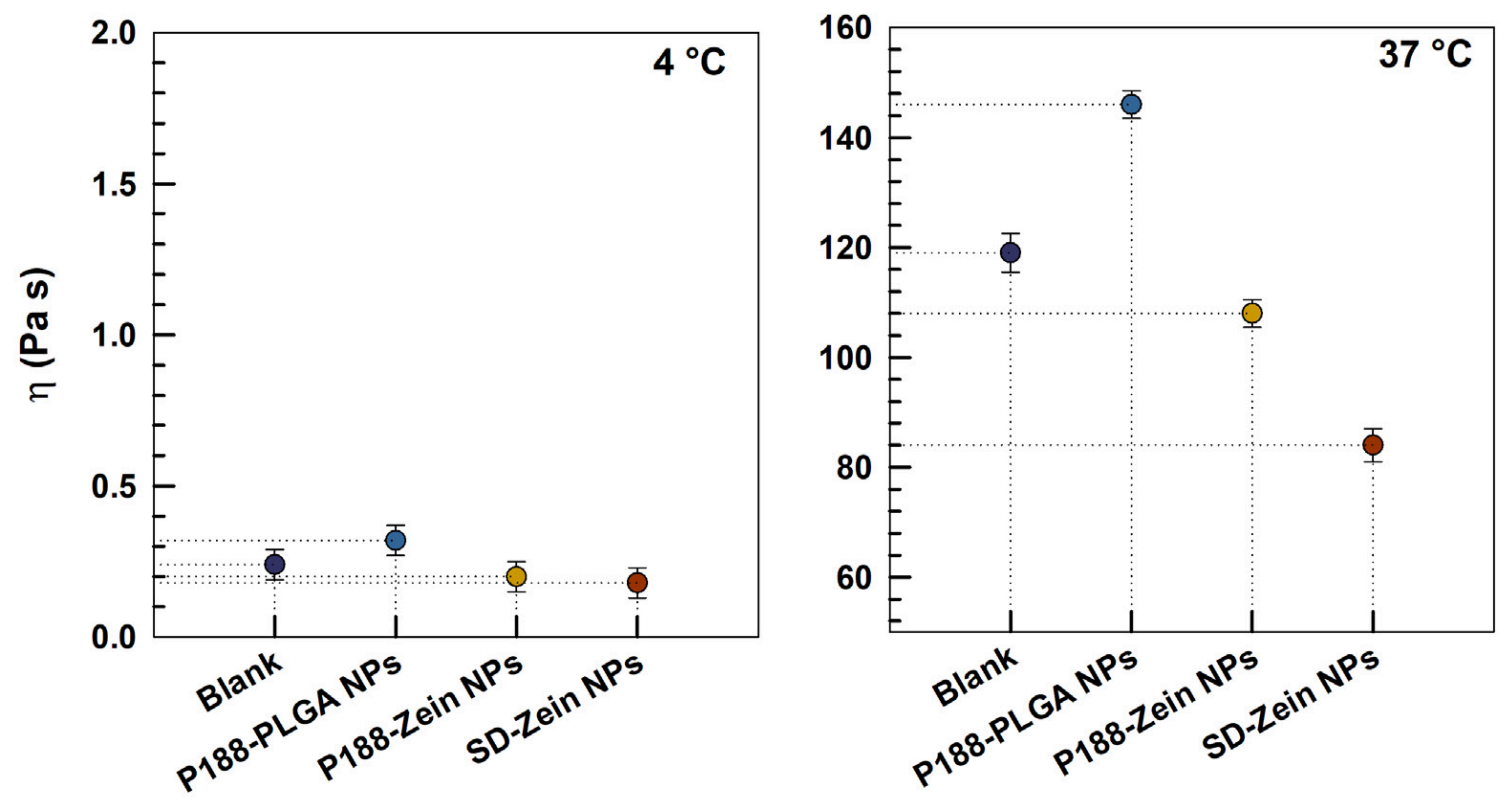
Viscosity of P908-based hydrogels at constant shear rate (1 s^−1^). Analyses were performed at 4 °C (storage temperature) and 37 °C (body temperature).

**Figure 3:**
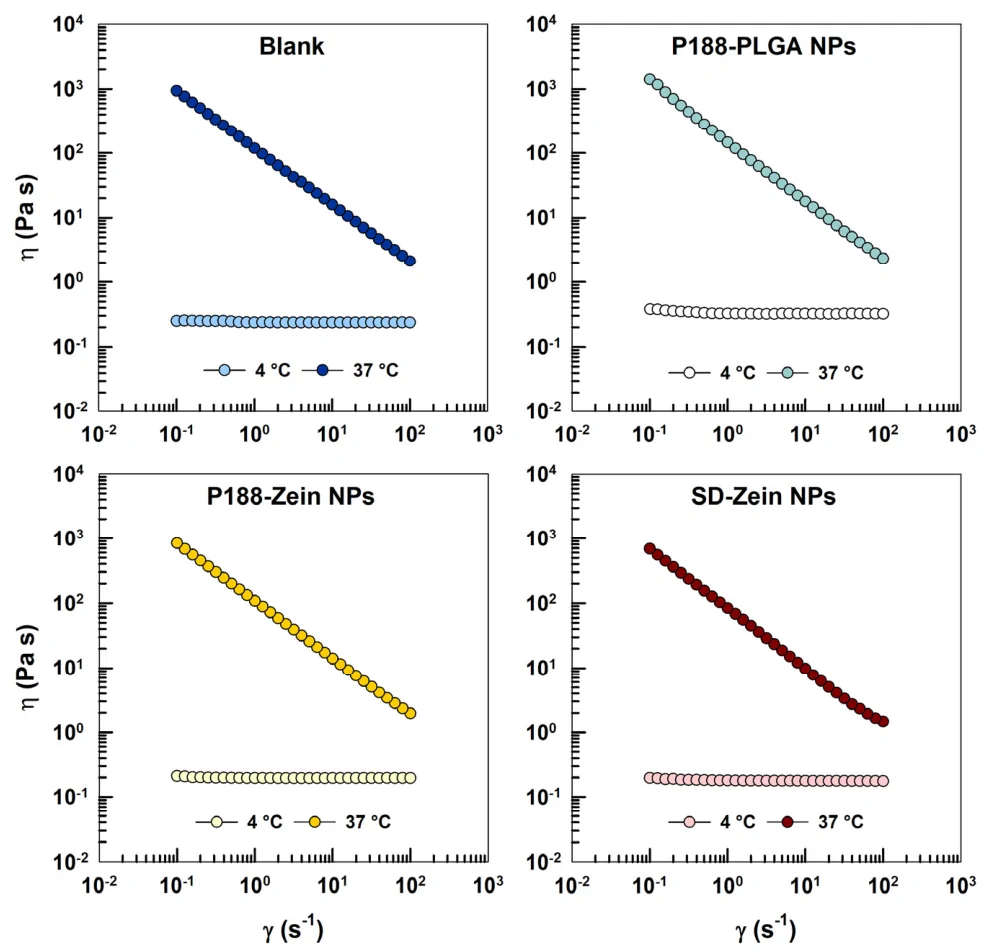
Steady-state shear viscosity of P908-based systems tested as an empty formulation or enriched with polymeric nanoparticles as a function of shear rate at 4 °C and 37 °C.

**Figure 4:**
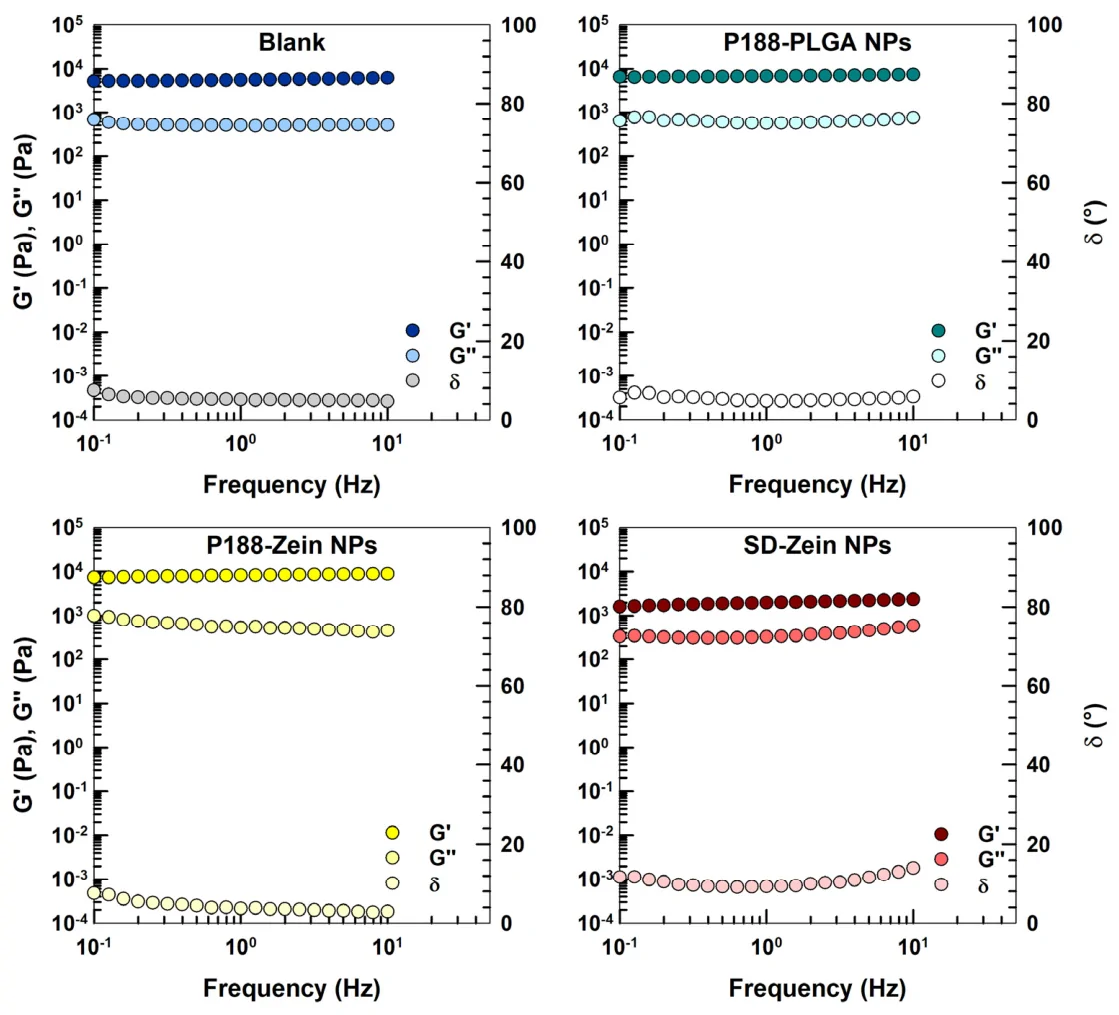
Effect of polymeric nanoparticles with different compositions on the viscoelastic moduli and phase angle of 30% (*w*/*w*) P908 hydrogels as a function of frequency at 37 °C.

**Figure 5:**
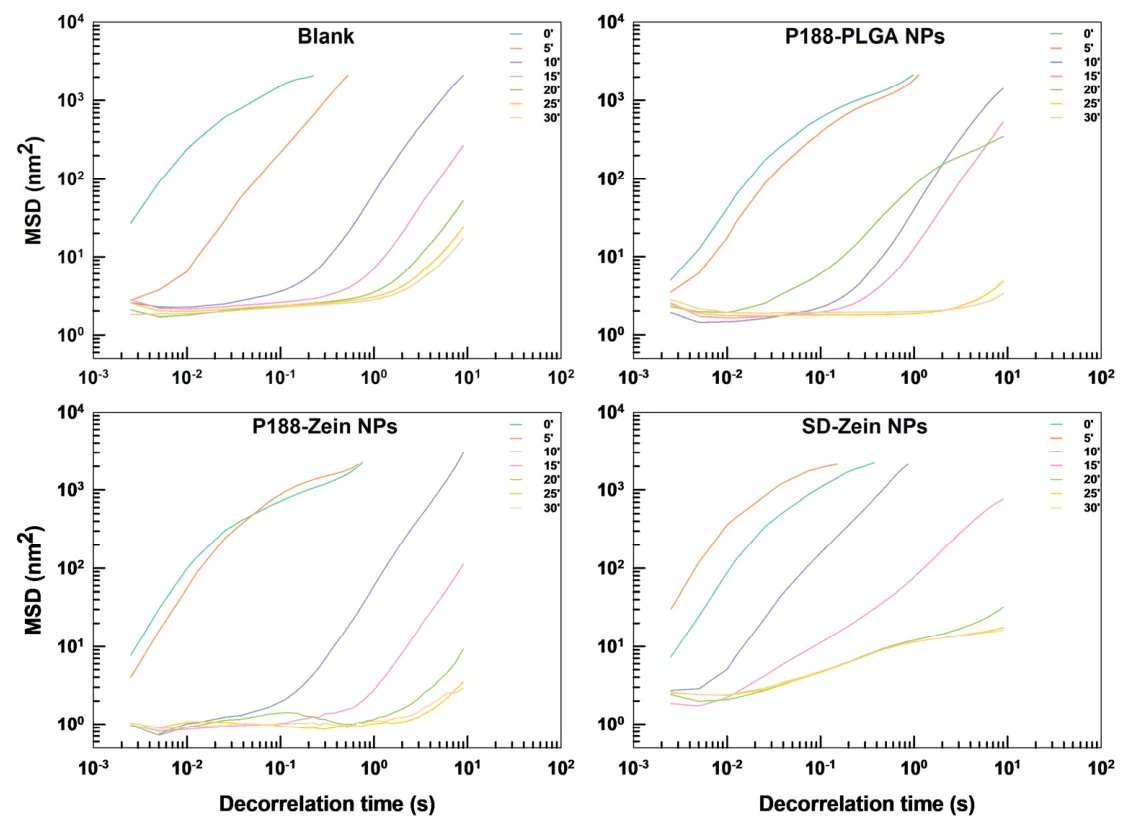
Mean square displacement (MSD) curves of P908-based systems, with or without incorporating the polymeric nanoparticles, as a function of the decorrelation time. The analysis was performed in the range between 4 and 37 °C.

**Figure 6:**
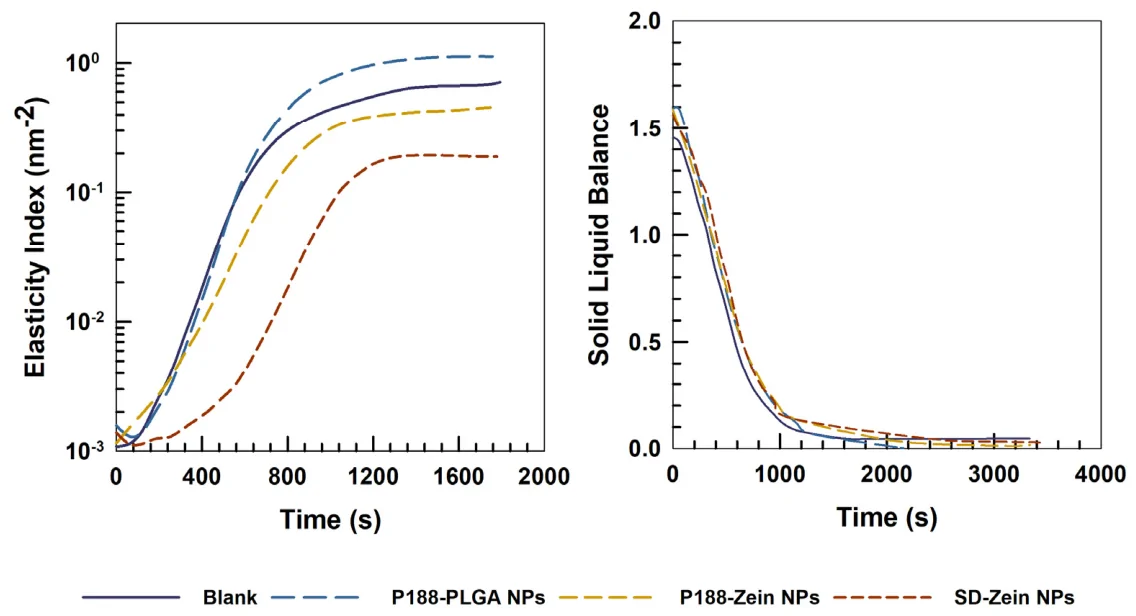
Elasticity Index (EI) and Solid–Liquid Balance (SLB) profiles of poloxamine 908 hydrogels as a function of time in the range of 4–37 °C.

**Figure 7:**
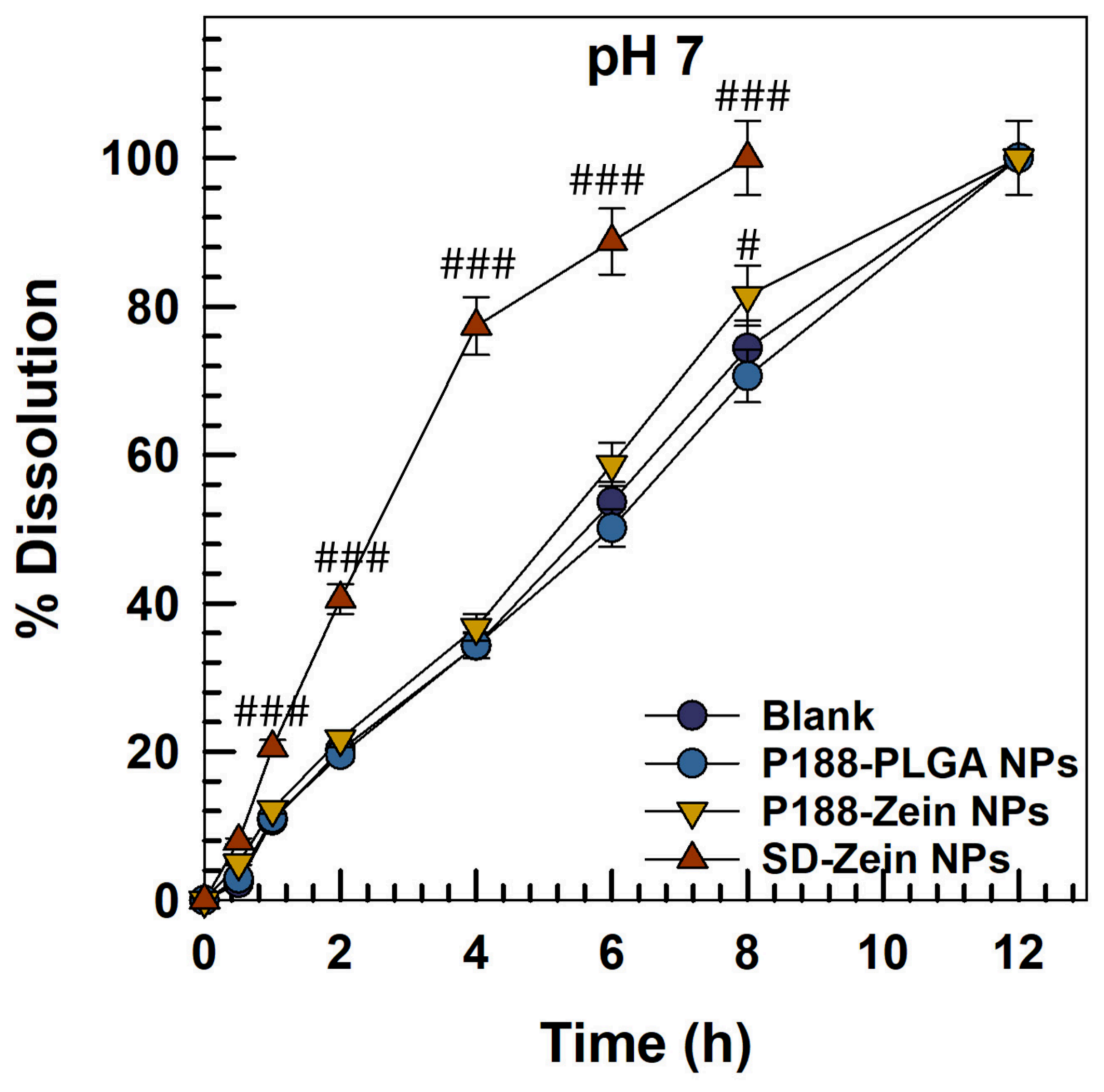
Effect of nanoparticle composition on the dissolution profiles of 30% *w*/*w* P908-based hydrogels at pH 7 and 37 °C as a function of time. Data are reported as mean ± SD of three independent experiments (n = 3). Statistical differences were evaluated by two-way repeated-measures ANOVA followed by Bonferroni multiple-comparisons test. **^#^** *p* < 0.05 and **^###^** *p* < 0.001 vs. the blank P908 hydrogel at the corresponding sampling time.

**Figure 8:**
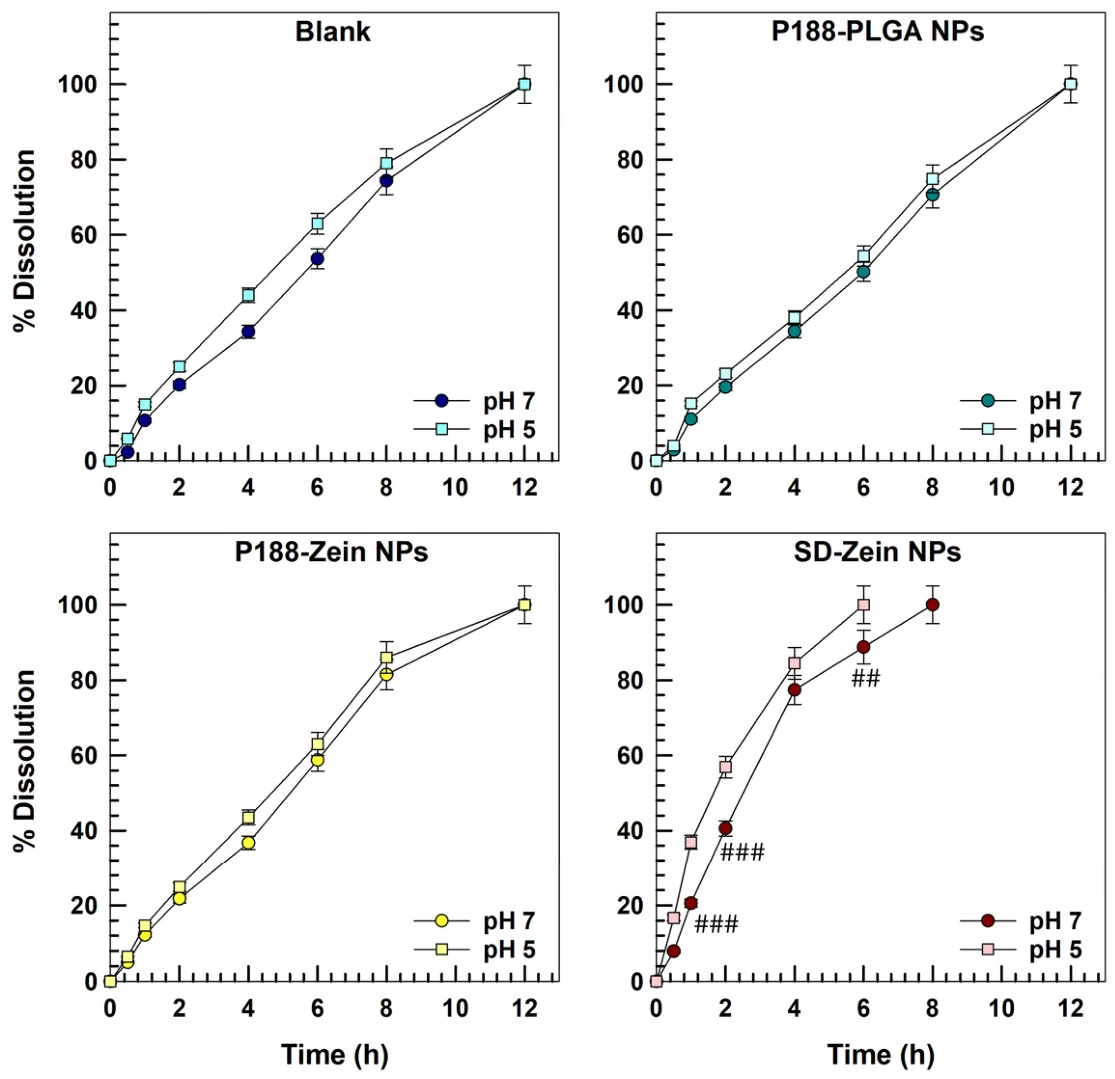
Effect of pH on the dissolution profiles of the different P908 formulations at 37 °C as a function of time. Data are reported as mean ± SD of three independent experiments (n = 3). Statistical differences were evaluated by two-way repeated-measures ANOVA followed by Bonferroni multiple-comparisons test. **^##^** *p* < 0.01 and **^###^** *p* < 0.001 vs. the corresponding nanocomposite hydrogel at the same sampling time and pH 5.0.

**Figure 9:**
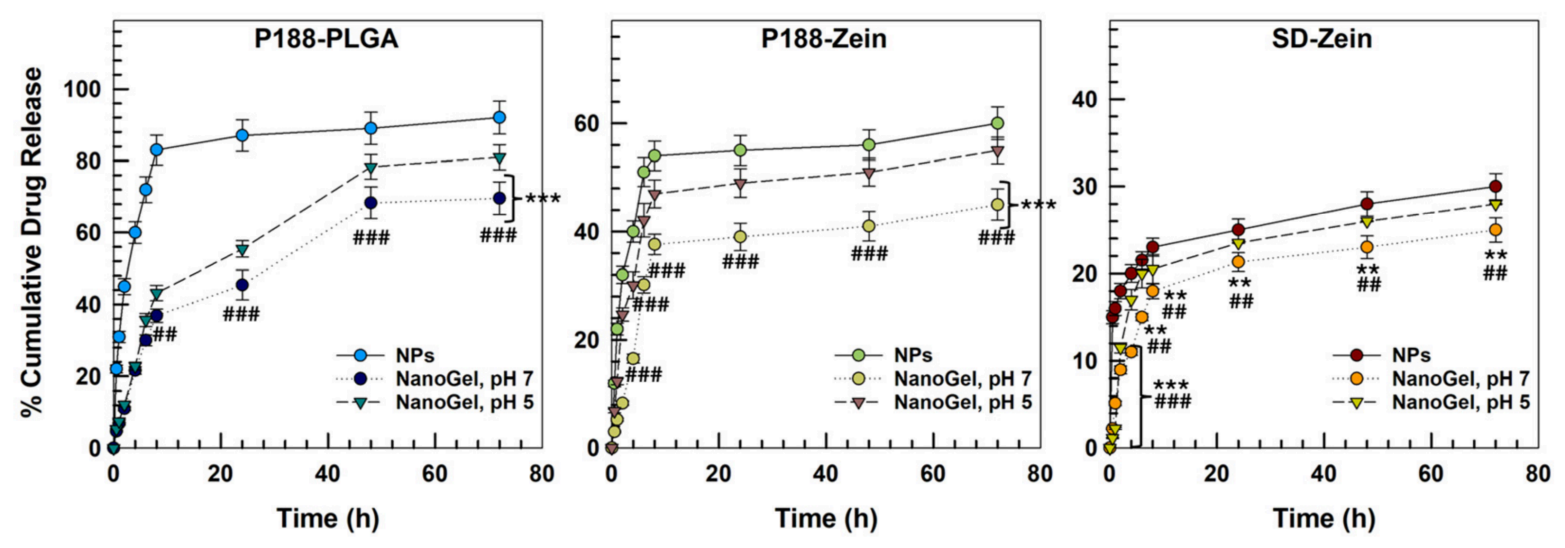
Release profiles of rutin-loaded P188-PLGA, P188-zein, and SD-zein nanoparticles at pH 7 and the corresponding nanogels at pH 7 and pH 5 reported as a function of time. Analyses were performed at 37 °C, and data are expressed as mean ± SD of three independent experiments (n = 3). Statistical differences were evaluated by two-way repeated-measures ANOVA followed by Bonferroni multiple-comparisons test. ** *p* < 0.01 and *** *p* < 0.001 vs. the corresponding NPs at the same sampling time; **^##^** *p* < 0.01 and **^###^** *p* < 0.001 vs. the corresponding nanocomposite hydrogel at the same sampling time and pH 5.0.

**Figure 10:**
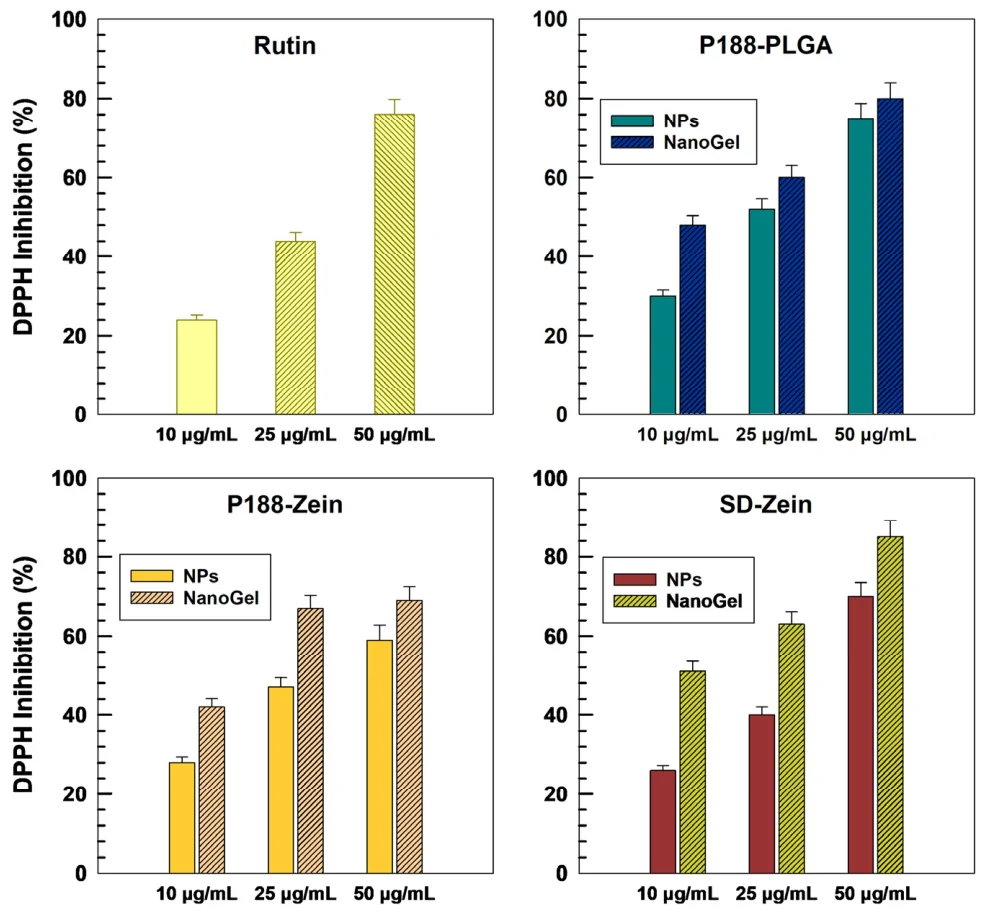
DPPH radical scavenging activity of rutin as a free form, entrapped within P188-PLGA, P188-zein and SD-zein nanoparticles (NPs) and contained in the corresponding P908-based nanocomposite hydrogels (NanoGel) at different rutin concentrations. Data are expressed as mean ± SD of three independent experiments (n = 3).

**Table 1:** Composition of the nanoparticle formulations selected for the development of nanocomposite hydrogels.

Formulation	Polymer Concentration	Stabilizer Concentration
P188 ^1^-PLGA ^2^	PLGA, 2.4 mg/mL	P188, 2.4% *w*/*v*
P188-zein	Zein, 2 mg/mL	P188, 2.5% *w*/*v*
SD ^3^-zein	Zein, 2 mg/mL	SD, 1.25% *w*/*v*

^1^ Poloxamer 188; ^2^ poly(D,l-lactide-co-glycolide); ^3^ sodium deoxycholate monohydrate.

**Table 2:** T_sol-gel_^1^ of the P908-based formulations.

P908-Based Hydrogel	T_sol-gel_ (°C)
Blank	26.4
P188-PLGA	28.5
P188-zein	28.8
SD-zein	33.9

^1^ Solution–gel transition temperature.

## Data Availability

Data will be made available on request.

## Supplementary Materials

The following supporting information can be downloaded at https://www.mdpi.com/article/10.3390/pharmaceutics18101204/s1, Figure S1: Schematic representation of the overall experimental workflow, including the preparation of polymeric nanoparticles, incorporation of the nanosystems into 30% (*w*/*w*) P908 hydrogels, and subsequent physicochemical, rheological, dissolution/release and antioxidant characterization; Figure S2: Effect of polymeric nanoparticles (NPs) with different compositions on the viscoelastic moduli and phase angle of 30% (*w*/*w*) P908 hydrogels as a function of temperature (10–50 °C). P908, Poloxamine 908; P188, Poloxamer 188; PLGA, poly(lactic-co-glycolic acid); SD, sodium deoxycholate; Figure S3: DPPH radical scavenging activity of empty P188-PLGA, P188-zein, and SD-zein nanoparticles (NPs) and the corresponding P908-based nanocomposite hydrogels (NanoGels) at different nanoparticle concentrations (10–50 μg/mL). The antioxidant activity of P908 alone was also evaluated at 5% (*w*/*w*). Data are expressed as the mean ± SD of three independent experiments. P908, Poloxamine 908; P188, Poloxamer 188; PLGA, poly(lactic-co-glycolic acid); SD, sodium deoxycholate; Table S1: Physico-chemical properties of the prepared nanoparticle formulations selected for the development of nanocomposite hydrogels. Each value is the mean of three different experiments ± standard deviation (SD); Table S2. Correlation coefficients (R^2^) of rutin-release kinetics from P908-based nanocomposite hydrogels at pH 7.0 according to different mathematical models; Table S3. Correlation coefficients (R^2^) of rutin-release kinetics from P908-based nanocomposite hydrogels at pH 5.0 according to different mathematical models.

## Abbreviations

The following abbreviations are used in this manuscript:

P908	Poloxamine 908
P407	Poloxamer 407
P188	Poloxamer 188
PLGA	Poly(lactic-co-glycolic acid)
SD	Sodium Deoxycholate
MSD	Mean Square Displacement
EI	Elasticity Index
SLB	Solid–Liquid Balance Parameter
