# Supplementary material for: Unlocking the Potential of Poloxamine 908 for Injectable Nanocomposite Hydrogels: Rheological Performance, Dissolution Behavior and Sustained Drug Release
Source: Pharmaceutics. 2026 Sep 22;18(10):1204. doi: 10.3390/pharmaceutics18101204 (PMC13708161; doi:10.3390/pharmaceutics18101204)
Supplement: Supplementary file 1 [file pharmaceutics-18-01204-s001.zip › pharmaceutics-4541123-supplementary.pdf]

pharmaceutics

MDPI

Article

Unlocking the Potential of Poloxamine 908 for Injectable Nanocomposite Hydrogels: Rheological Performance, Dissolution Behavior and Sustained Drug Release

Elena Giuliano [1,2], Agnese Gagliardi [1,2], Emanuela Longo [1], Valeria Gaetano [1], Shabir Ahmad [1,3] and Donato Cosco [1,2*]

[1] Department of Health Sciences and [2] "AGreenFood" Research Center, University of Catanzaro "Magna Græcia", Campus Universitario "S. Venuta", I-88100 Catanzaro, Italy; elena.giuliano@unicz.it, gagliardi@unicz.it, emanuela.longo@unicz.it, valeria.gaetano@unicz.it

[3] Department of Chemical, Materials, and Industrial Production Engineering (DICMaPI), University of Naples Federico II, 80125, Naples, Italy; shabir.ahmad@unina.it

* Correspondence: donatocosco@unicz.it

Supplementary Materials

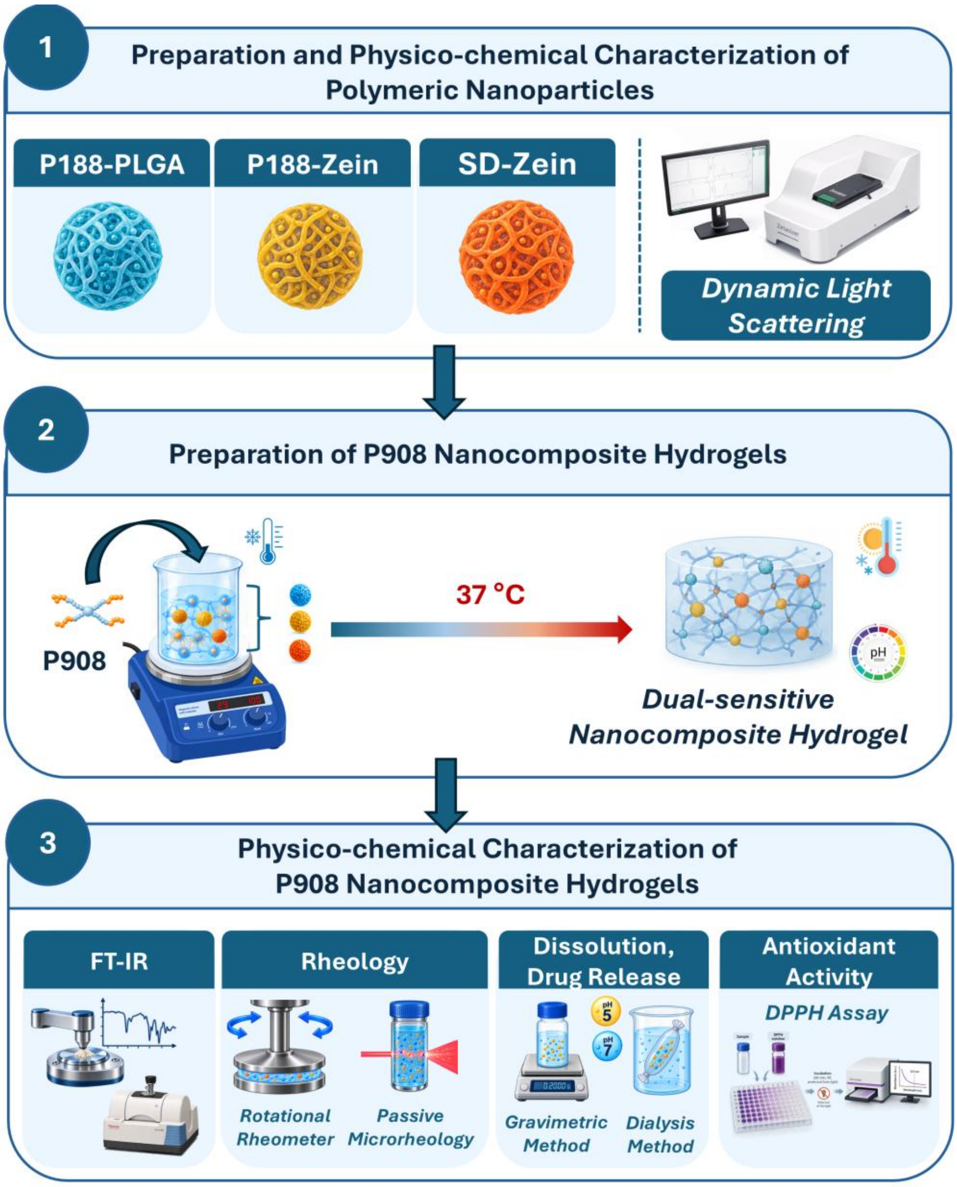

Figure S1: Schematic representation of the overall experimental workflow, including the preparation of polymeric nanoparticles, incorporation of the nanosystems into 30% (w/w) P908 hydrogels, and subsequent physicochemical, rheological, dissolution/release and antioxidant characterization.

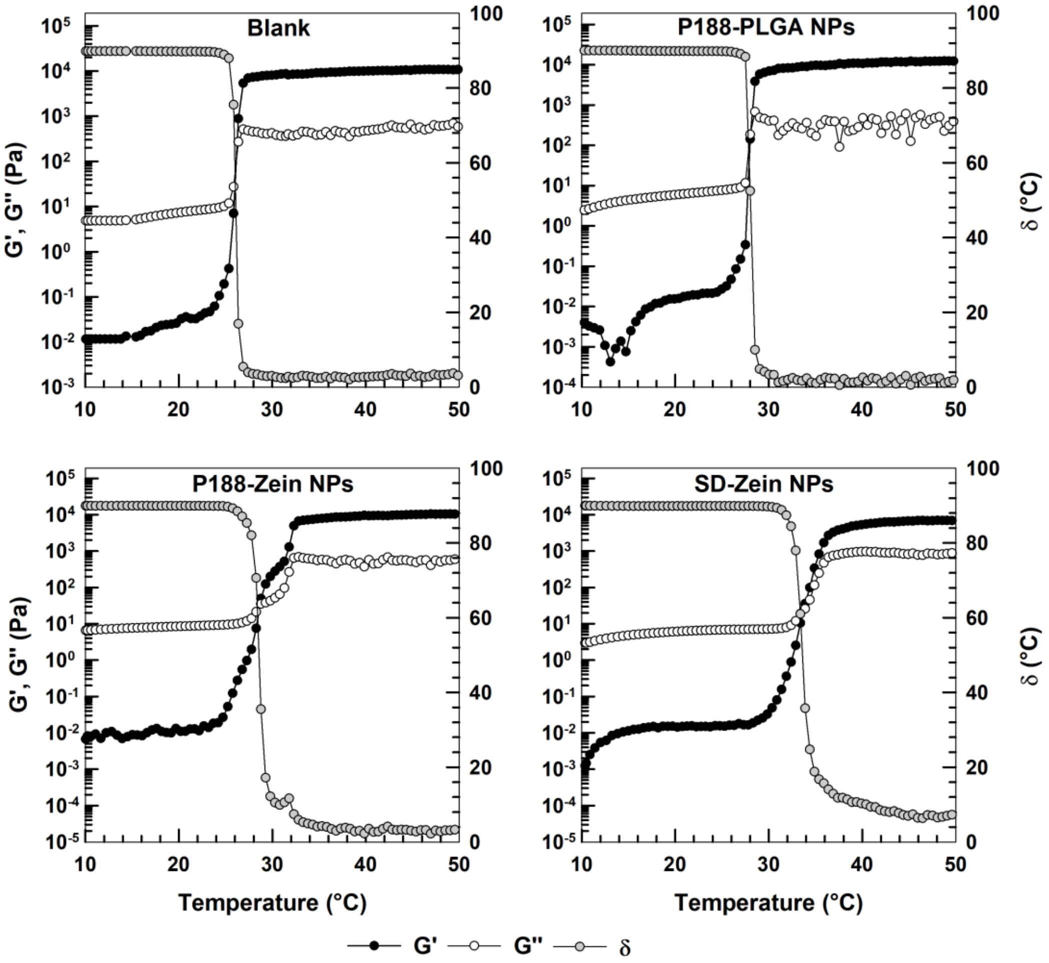

Figure S2. Effect of polymeric nanoparticles (NPs) with different compositions on the viscoelastic moduli and phase angle of 30% (w/w) P908 hydrogels as a function of temperature (10–50 °C). P908, Poloxamine 908; P188, Poloxamer 188; PLGA, poly(lactic-co-glycolic acid); SD, sodium deoxycholate.

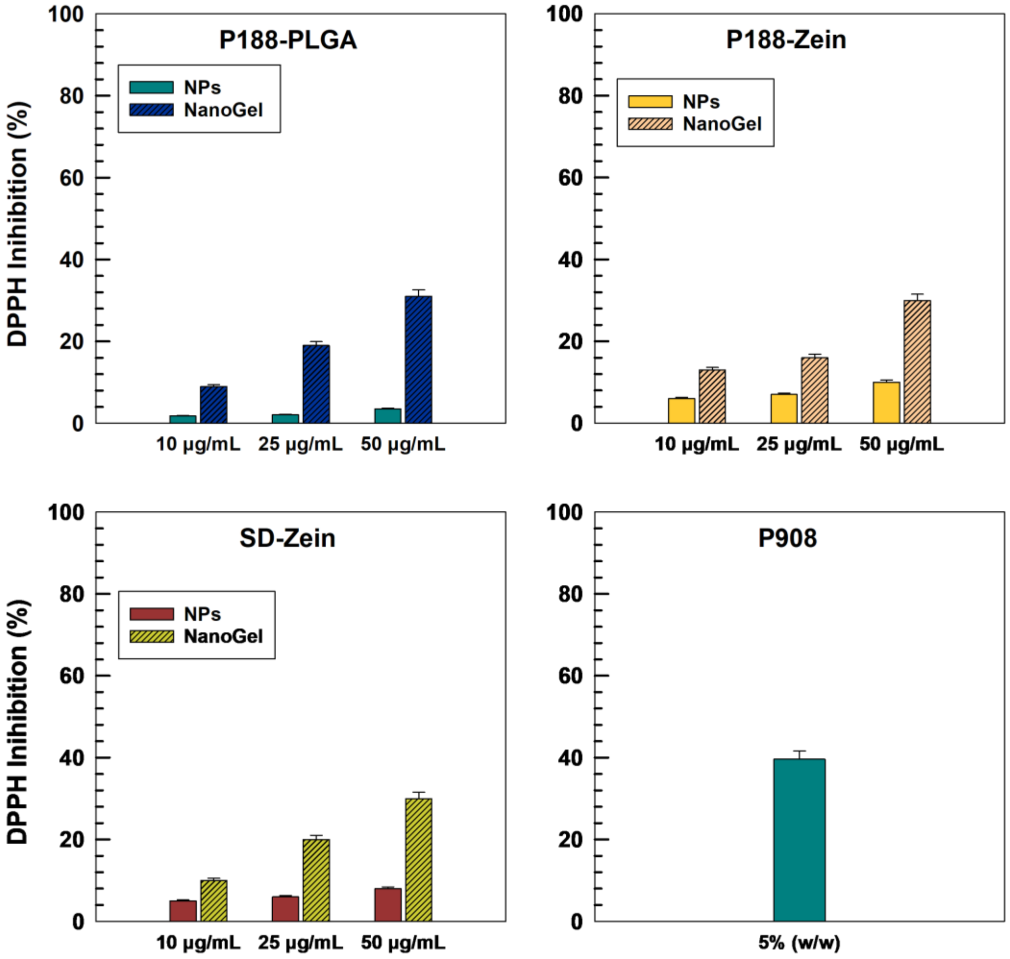

Figure S3. DPPH radical scavenging activity of empty P188-PLGA, P188-Zein, and SD-Zein nanoparticles (NPs) and the corresponding P908-based nanocomposite hydrogels (NanoGels) at different nanoparticle concentrations (10–50 µg/mL). The antioxidant activity of P908 alone was also evaluated at 5% (w/w). Data are expressed as the mean ± SD of three independent experiments). P908, Poloxamine 908; P188, Poloxamer 188; PLGA, poly(lactic-co-glycolic acid); SD, sodium deoxycholate.

Table S1. Physico-chemical properties of the prepared nanoparticle formulations selected for the development of nanocomposite hydrogels. Each value is the mean of three different experiments ± standard deviation (SD).

Formulation	Mean sizes ± SD (nm)	Polydispersity Index ± SD	Zeta Potential ± SD (mV)
P188[1]-PLGA[2]	120 ± 1	0.08 ± 0.01	-22 ± 2
P188-Zein	121 ± 2	0.15 ± 0.02	23 ± 1
SD[3]-Zein	110 ± 1	0.11 ± 0.01	-33 ± 2

[1] poloxamer 188; [2] poly(D,l-lactide-co-glycolide); [3] sodium deoxycholate monohydrate.

Table S2. Correlation coefficients (R^2) of rutin release kinetics from P908-based nanocomposite hydrogels at pH 7.0 according to different mathematical models.

P908-based Hydrogel	Zero order	First order	Higuchi	Korsmeyer-Peppas	Hixson-Crowel
P188-PLGA	0.98	0.95	**0.99**	0.88	0.97
P188-Zein	0.97	0.90	**0.98**	0.85	0.96
SD-Zein	0.94	0.95	**0.98**	0.89	0.95

Table S3. Correlation coefficients (R^2) of rutin release kinetics from P908-based nanocomposite hydrogels at pH 5.0 according to different mathematical models.

P908-based Hydrogel	Zero order	First order	Higuchi	Korsmeyer-Peppas	Hixson-Crowel
P188-PLGA	0.94	0.97	**0.98**	0.87	0.96
P188-Zein	0.93	0.96	**0.98**	0.89	0.95
SD-Zein	0.87	0.92	**0.96**	0.93	0.88
